# Energy Deficiency and Misdistribution Leads to Disrupted Formation in Grain Yield and Rice Quality

**DOI:** 10.3390/ijms252312751

**Published:** 2024-11-27

**Authors:** Yiding Wang, Guangyan Li, Jiaying Ma, Haoran Su, Wenfei Hu, Junjiang Lin, Weimeng Fu, Yvxiang Zeng, Longxing Tao, Guanfu Fu, Jie Xiong, Tingting Chen

**Affiliations:** 1 College of Life Sciences and Medicine, Zhejiang Sci-Tech University, Hangzhou 310018, China; wang_1ding@163.com (Y.W.); suhaoran0412@163.com (H.S.); 15593823808@163.com (W.H.); 18022557884@163.com (J.L.); 2 State Key Laboratory of Rice Biology and Breeding, China National Rice Research Institute, 359 Tiyuchang Road, Hangzhou 310006, China; gyli@yzu.edu.cn (G.L.); majiaying1108@163.com (J.M.); fuwmeng@163.com (W.F.); zengyvxiang@caas.cn (Y.Z.); taolongxing@caas.cn (L.T.); fuguanfu@caas.cn (G.F.); 3 Agricultural College, Yangzhou University, Yangzhou 225009, China

**Keywords:** grain yield, rice quality, carbohydrate accumulation, energy metabolism

## Abstract

With the progress of society and the improvement of agricultural scientific technology, the single focus on high yield for rice production has gradually shifted to high quality. Coordinated development of grain yield and rice quality has become a core issue for researchers, and the underlying mechanisms remain to be solved. Two varieties, Zhongzheyou1 (ZZY1) and Zhongzheyou8 (ZZY8), were used as study materials under field conditions. The yield of ZZY1 was higher than that of ZZY8, which was mainly characterized by a higher seed-setting rate and grain weight. The rice quality of ZZY8 was better than that of ZZY1, primarily due to lower chalkiness and a higher head rice rate. The total dry matter weight of ZZY1 was lower than that of ZZY8, but the proportion of panicle dry matter weight or nonstructural carbohydrate to the total in the former was higher than that of the latter. The maximum grain-filling rate, average grain-filling rate, and key enzyme activities of ZZY1 were significantly higher than those of ZZY8, while the active grain-filling period was shorter than that of ZZY8. Furthermore, the ATP/ATPase content and energy charge values in the grains of ZZY1 were higher than those of ZZY8 at the early grain-filling stage. Transcriptome analysis showed that carbohydrate and energy metabolism were the main ways affecting the yield and quality of the two varieties. The energy production of ZZY1 was insufficient to simultaneously supply the needs thus leading to the discordant formation in its grain yield and rice quality formation.

## 1. Introduction

China is the country with the largest rice demand in the world, but the impact of increasing population, decreasing land, and agricultural resource shortages on rice production is increasing. According to the “National Population Development Plan (2016–2030)”, China’s population is expected to reach a peak of 1.45 billion in 2030. Therefore, in order to ensure China’s food security, it is necessary to significantly increase rice yield on the existing basis [1,2,3]. The yield of rice is composed of panicle number, grains per panicle, seed setting rate, and grain weight, which restrict each other and jointly determine the final yield level [4]. Efforts have been made to improve grain yield, and a large amount of work on the physiological foundation for high yield in rice production has also been done by previous researchers [5,6,7,8].

In the past, the goal of rice production in our country was mainly high yield in order to ensure the basic needs of people’s lives, while the pursuit of high-quality rice started later. Generally, high-quality rice varieties often have the problems of low yield and poor adaptability, and the contradiction between “high yield” and “high quality” still exists widely [9,10]. As the economy develops and the social population’s living standards steadily improve in recent years, people’s demand for high-quality rice has increased. The high-quality rice production has developed rapidly under the condition that the yield basically meets the demand due to the continuous growth of rice yield since the 21st century [11,12]. The number of high-quality rice varieties has increased significantly, and rice production has been optimized. Among the ten rice varieties with the largest planting area in China in 2020, eight of them were high-quality varieties. Therefore, the goal of rice production at the current stage should be to improve rice yield and quality through variety selection and optimization of cultivation and management measures to achieve the coordinated development of grain yield and rice quality [13,14,15]. The focus should be on further improving the rice quality of high-yield rice varieties.

Some research results have been acquired on grain yield performance and rice quality changes under optimized agronomic techniques [6,13,14,15]. Studies have shown that the characteristics of high-yield and high-quality japonica rice varieties mainly showed a grain yield range of 8.35~9.16 t hm^−2^ and a food taste score range of 60~74 [16]. Research has also demonstrated that japonica rice varieties with excellent eating taste and high yield were characterized by large panicles with a large quantity of spikelets, low grain weight, and strong dry matter and nitrogen accumulation ability in the middle and late growth periods [17]. These varieties were also characterized by high leaf photosynthetic rate, high nitrogen absorption rate, and high activities of key enzymes involved in carbon and nitrogen metabolism, such as sucrose phosphate synthase, sucrose synthase, glutamate synthase, and glutamine synthase, all of which would promote the efficient transfer and reuse of carbon and nitrogen from the vegetative organs to the panicle [18]. However, the obstacle to synergistically improving grain yield and the underlying physiological mechanisms were unclear.

Energy is the foundation of all living activities, and the growth and development of all organisms depend on a constant mechanism of energy transfer [19]. Plants can obtain a significant amount of ATP through photosynthesis in their leaves [20]. The energy in the seeds mainly comes from the decomposition of assimilates unloaded from the source end through cellular respiration, producing ATP and other forms of energy for cellular metabolism. Previous studies have shown that the process of rice yield formation is one of dry matter accumulation and transport, and a substantial amount of energy is consumed during material transport [21,22,23]. Insufficient energy will inhibit pollen germination and pollen tube elongation under heat stress, hinder assimilate transport and metabolism, lead to reduced seed setting, inadequate grain filling, and ultimately constrain yield and quality formation [24]. Generally, it can be seen that the formation of crop yield and quality is a process that requires high energy consumption, which could pertinently explain the contradictoriness in simultaneously obtaining high yield and high quality. We supposed that energy deficiency was the main reason that led to the difficulty in collaborative enhancement for grain yield and rice quality. The assumption was verified in this current study by using two experimental materials, Zhongzheyou1 (ZZY1, higher grain yield and poorer rice quality) and Zhongzheyou8 (ZZY8, lower grain yield and better rice quality). Grain yield and rice quality were compared, and the related processes such as dry matter accumulation and carbohydrate metabolism were observed. The role of energy metabolism in the generation, utilization, and distribution of energy in the formation of rice yield and quality was primarily focused. Furthermore, transcriptome analysis was also performed to better understand the relevant mechanisms underlying the synergistic formation of grain yield and rice quality.

## 2. Results

### 2.1. Grain Yield, Its Components, and Rice Quality

**Figure 1:**
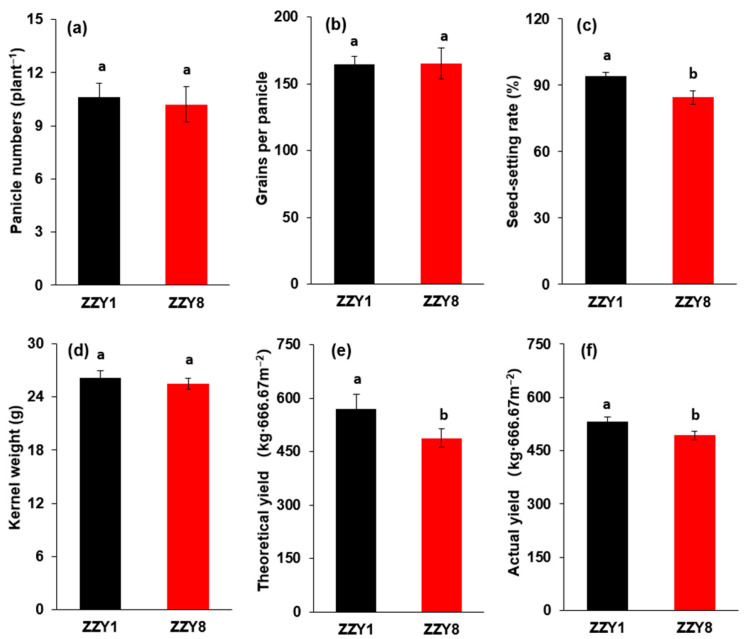
Grain yield and its components of two rice varieties. (**a**) Panicle numbers; (**b**) grain numbers of each panicle; (**c**) seed-setting rate; (**d**) kernel weight; (**e**) theoretical yield calculated by yield components; (**f**) actual grain yield. ZZY1 and ZZY8 indicate rice varieties, Zhongzhouyou1 and Zhongzhouyou8, respectively; error bars denote ± standard deviation (*n* = 3); different letters between varieties indicate significant differences at the 0.05 probability level.

As shown in Figure 1, ZZY1 has a theoretical yield of 8.6 t ha^−1^, which is 17.1% higher than the theoretical yield of 7.3 t ha^−1^ of ZZY8, and the difference was significant at the *p* = 0.05 level. However, the actual yield increase was reduced to only 7.9%, and the difference was not significant. In terms of yield components, ZZY1 has both a higher number of panicles per plant and a higher 1000-grain weight compared to ZZY8, although it has a lower number of grains per panicle. However, those differences were all not significant. The seed-setting rate of ZZY1 was significantly higher than that of ZZY8, and the increment was 11.5%.

**Figure 2:**
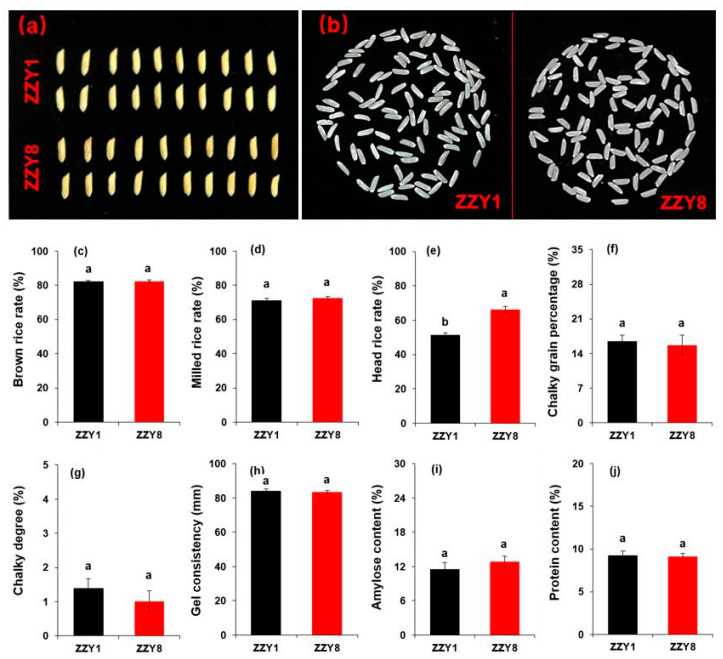
Grain appearance and rice quality parameters. (**a**) Unshelled kernel; (**b**) milled rice; (**c**) brown rice rate; (**d**) milled rice rate; (**e**) head rice rate; (**f**) chalky grain rate; (**g**) chalky degree; (**h**) gel consistency; (**i**) amylose content; (**j**) protein content. ZZY1 and ZZY8 indicate rice varieties, Zhongzhouyou1 and Zhongzhouyou8, respectively; error bars denote ± standard deviation (*n* = 3); different letters between varieties indicate significant differences at the 0.05 probability level.

Unshelled kernel and milled rice appearance are shown in Figure 2a,b. Rice quality parameters, including brown rice rate, milled rice rate, gel consistency, and protein content, showed no significant difference between varieties (Figure 2c,d,f–j). However, ZZY8 has a significantly higher head-milled rice rate than ZZY1 (Figure 2e). The chalkiness degree and chalky grain rate of ZZY1 are both higher than those of ZZY8, with an increase of 28.6% and 6.3%, respectively, although the difference was not significant (Figure 2f,g). The amylose content of ZZY1 was slightly lower than that of ZZY8, but the difference was not significant (Figure 2i).

### 2.2. Plant Height and Dynamic Changes of Tillering

**Figure 3:**
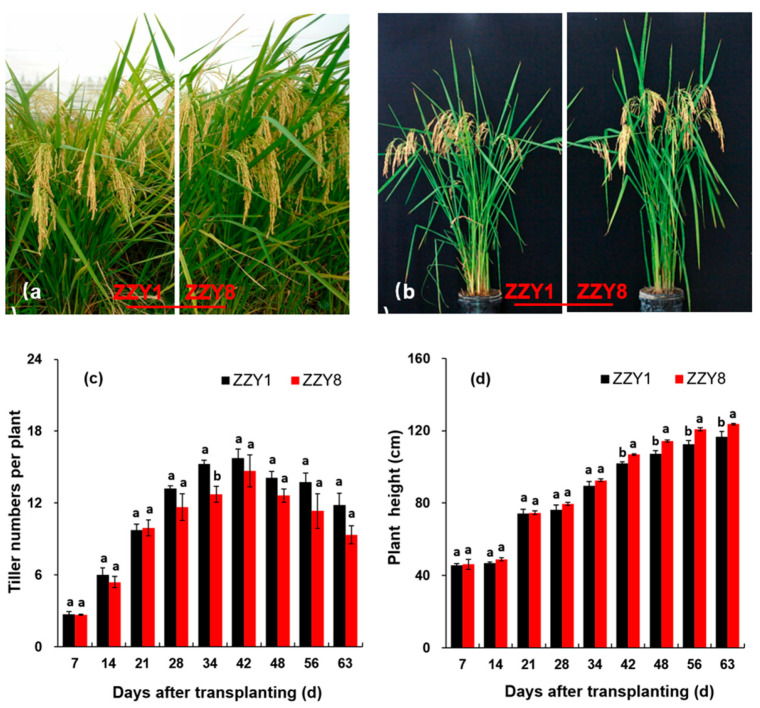
Plant morphology and the dynamic change of tiller numbers and plant height of rice plants. (**a**) Rice morphology at field condition; (**b**) plant morphology in pot experiment; (**c**) tiller numbers; (**d**) plant height. ZZY1 and ZZY8 indicate rice varieties, Zhongzhouyou1 and Zhongzhouyou8, respectively; error bars denote ± standard deviation (*n* = 3); different letters between varieties indicate significant differences at the 0.05 probability level.

Field performance is exhibited in Figure 3a,b. During the entire determination period, the plant height of ZZY8 was consistently taller than that of ZZY1 (Figure 3a,b,d). The plant height growth rate of both varieties reached the fastest between 14–21 days after transplanting, with increases of 36.7% in ZZY1 and 34.6% in ZZY8, respectively. At 56 days after transplanting, the difference in plant height between the two varieties reached its maximum. At 63 days after transplanting, the plant heights of the two varieties were 116.5 cm and 123.7 cm, respectively, with ZZY8 being 5.82% taller than ZZY1. Both varieties reached the tiller number peak at 42 days after transplanting (Figure 3c). Throughout the whole determination period, the tiller numbers of ZZY1 were higher than ZZY8, except for 21 days after transplanting. The greatest difference occurred at 63 days after transplanting, when the tiller numbers of ZZY1 were 21.1% higher than those of ZZY8.

### 2.3. Leaf Chlorophyll Contents and Photosynthetic Rate

**Figure 4:**
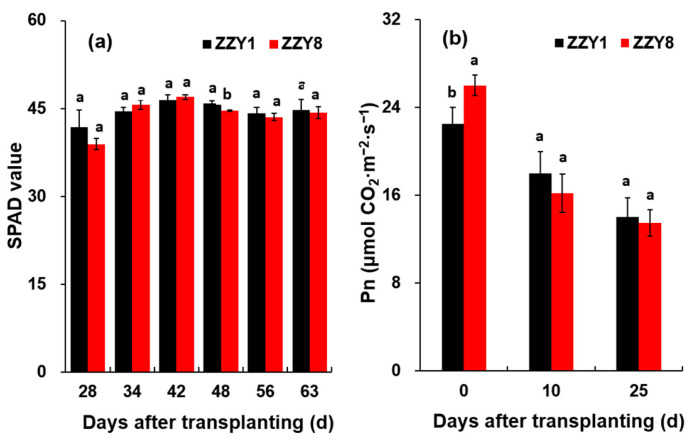
The chlorophyll content index (SPAD value) and net photosynthetic rate of rice leaf. (**a**) SPAD value; (**b**) net photosynthetic rate. ZZY1 and ZZY8 indicate rice varieties, Zhongzhouyou1 and Zhongzhouyou8, respectively; error bars denote ± standard deviation (*n* = 3). Different letters between varieties indicate significant differences at the 0.05 probability level.

The chlorophyll contents of both varieties generally followed a trend of increasing and then decreasing, as demonstrated in Figure 4a. Except for some certain dates, the chlorophyll content of ZZY1 was slightly higher than that of ZZY8, although the difference between varieties was not obvious. The net photosynthetic rate of the flag leaf showed a gradual declining trend, with the highest value at the heading stage (Figure 4b). The net photosynthetic rate of the flag leaf of ZZY8 was significantly higher than that of ZZY1 at the heading stage, while it was reversed at 10 and 25 days after heading, although the difference was not significant.

### 2.4. Dry Matter, NSC Production, and Translocation

**Figure 5:**
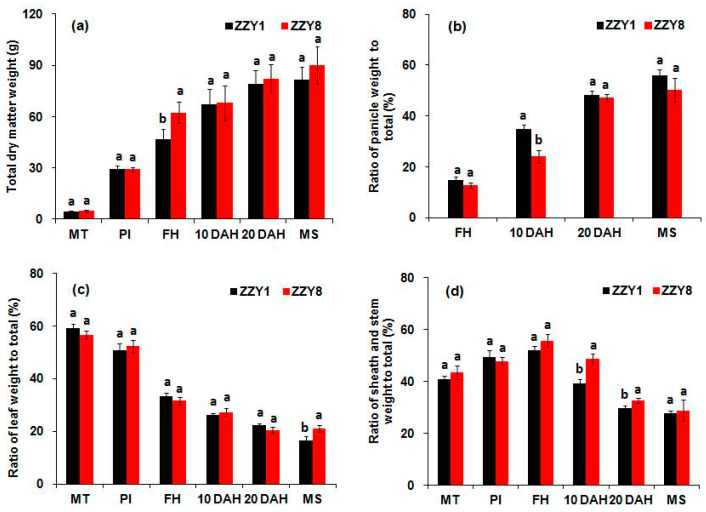
Total dry matter accumulation and allocation in rice plants. (**a**) Total dry weight; (**b**) ratio of panicle weight to total dry weight; (**c**) ratio of leaf weight to total dry weight; (**d**) ratio of sheath and stem weight to total dry weight. MT, PI, FH, MS, and DAH denote midtillering, panicle initiation, full-heading, maturity stage, and days after heading. ZZY1 and ZZY8 indicate rice varieties, Zhongzhouyou1 and Zhongzhouyou8, respectively; error bars denote ± standard deviation (*n* = 3). Different letters between varieties at the same determination time indicate significant differences at the 0.05 probability level.

The total dry matter weight of ZZY1 and ZZY8 gradually increased as growth progressed (Figure 5a). The dry matter weight of ZZY8 was slightly higher than that of ZZY1 at most periods, although the difference was not significant. The proportion of panicle dry matter to total dry matter in ZZY1 was higher than ZZY8 from heading to maturity, whereas the difference at 10 DAH was significant (Figure 5b). The proportion of leaf dry matter to total dry matter showed a gradually decreasing trend (Figure 5c). There was no clear change pattern in the differences between the two varieties. The proportion of stem and sheath dry matter to total dry matter first increased and then decreased with the development growth (Figure 5d). The ratio in ZZY8 was higher than that in ZZY1 at all dates except for the panicle differentiation stage, and the difference was significant at 10 and 20 DAH.

**Figure 6:**
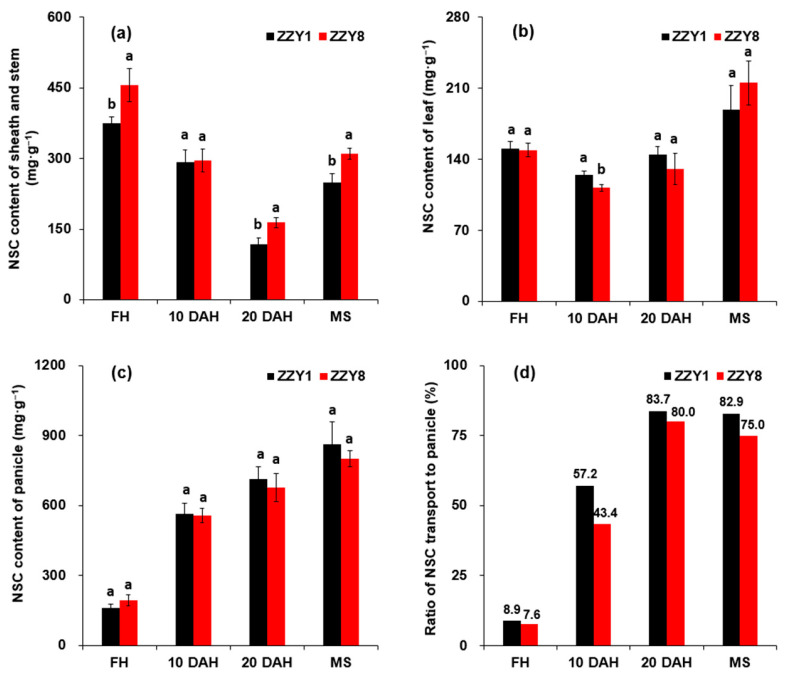
The nonstructural carbohydrate (NSC) content accumulation and allocation in rice plants. (**a**) NSC content in sheath and stem; (**b**) NSC content in leaf; (**c**) NSC content in panicle; (**d**) ratio of NSC content in panicle to the total. FH, MS, and DAH denote full heading, maturity stage, and days after heading; ZZY1 and ZZY8 indicate rice varieties, Zhongzhouyou1 and Zhongzhouyou8, respectively; error bars denote ± standard deviation (*n* = 3). Different letters between varieties at the same determination time indicate significant differences at the 0.05 probability level.

The nonstructural carbohydrate (NSC) content in the stem, sheath, and leaves of ZZY1 and ZZY8 first decreased and then increased as the growth period proceeded, with the highest content in the sheath at the heading stage and in the leaves at maturity (Figure 6a,b). The NSC content in the sheath of ZZY8 was consistently higher than that of ZZY1, where the difference was significant at most dates (Figure 6a). The NSC content in the leaves of ZZY1 was slightly higher than that of ZZY8 from the heading to 20 DAH (Figure 6b). However, it was reversed at maturity, and the difference between the varieties was not significant. Except for the heading stage, the NSC content in the panicles of ZZY8 was lower than that of ZZY1 at 10 DAH, 20 DAH, and maturity (Figure 6c). The decrease ranged from 1.5% to 7.2%, and the difference was not significant. The proportion of panicle NSC to total NSC in ZZY1 was higher than in ZZY8 throughout the entire grain-filling period, and the difference reached the largest at 10 DAH (Figure 6d).

### 2.5. Characteristics of Grain-Filling

**Figure 7:**
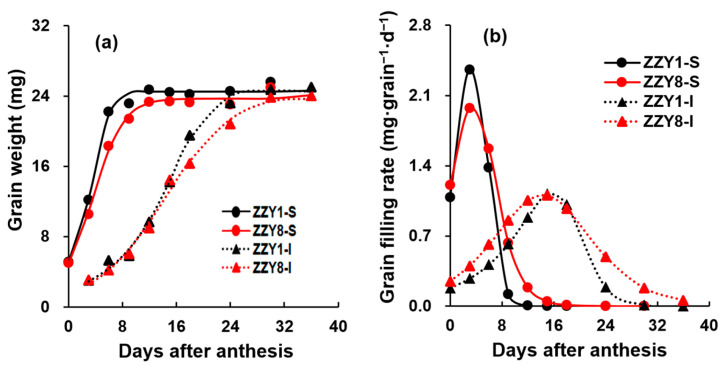
The grain weight and grain-filling rate dynamics of rice. (**a**) Grain weight; (**b**) grain-filling rate; ZZY1 and ZZY8 indicate rice varieties, Zhongzhouyou1 and Zhongzhouyou8, respectively; S and I indicate superior and inferior spikelets.

The grain-filling process of the two varieties estimated using Richards’ growth equation is shown in Figure 7. The R2 values derived from the equation W = A/(1+Be-kt)1/N were mostly above 0.95, indicating a high fitting degree, and the grain-filling process conformed well to the Richards’ growth model. The final grain weights of ZZY1 were higher than ZZY8, with a difference of 0.12 g in superior grains and 0.10 g in inferior grains (Figure 7a). Figure 7b presents the grain-filling dynamics, where the grain weight of ZZY1 was higher than that of ZZY8 regardless of superior or inferior grains throughout the whole course. The superior grains of both varieties reached their maximum grain-filling rates three days after flowering and then declined rapidly (Figure 7b). The grain-filling rate of ZZY1 was obviously higher than ZZY8 during the first six days, and the former was notably higher than the latter. In comparison, the grain-filling rates of inferior grains increased slowly, reached their maximum at 15 days after anthesis, and then gradually declined in both varieties, especially for ZZY8.

ZZY1 had higher mean grain-filling rates than ZZY8 for both superior and inferior grains (Table 1). In terms of the active grain-filling period, the superior grains of ZZY1 lasted for 10.6 days, while those of ZZY8 lasted for 14.5 days. For the inferior grains, ZZY1 and ZZY8 lasted 23.7 days and 30.8 days, respectively. The number of days to reach the maximum grain-filling rate of ZZY1 exceeded ZZY8 in both superior and inferior grains. A similar tendency was found in changes in the maximum grain-filling rate.

### 2.6. Carbohydrate Content and Key Enzymes Related to Grain-Filling

**Figure 8:**
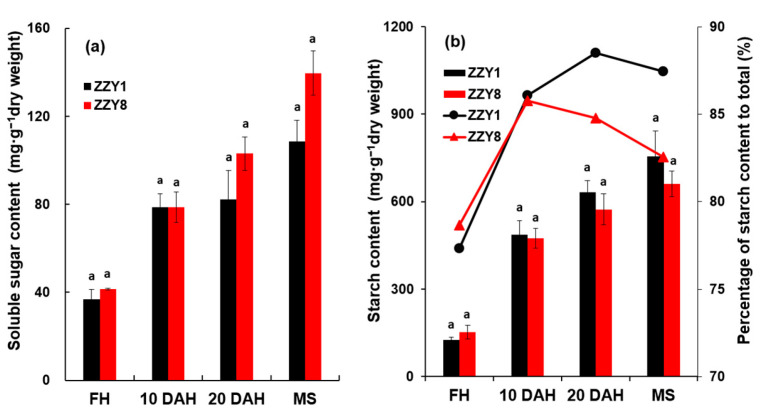
Soluble sugar and starch content in grains of rice. FH, MS, and DAH denote full heading, maturity stage, and days after heading. (**a**) Soluble sugar content; (**b**) starch content. FH, MS, and DAH denote full heading, maturity stage, and days after heading; ZZY1 and ZZY8 indicate rice varieties, Zhongzhouyou1 and Zhongzhouyou8, respectively; error bars denote ± standard deviation (*n* = 3); different letters between varieties at the same determination time indicate significant differences at the 0.05 probability level.

As shown in Figure 8, the content of soluble sugars in the grains showed a gradually increasing trend from the heading stage to maturity. The content of soluble sugars in ZZY1 grains was lower than that in ZZY8 at most determination stages, and the largest difference was observed at maturity (Figure 8a). The starch content in the grains also showed a gradually increasing trend. The starch content in ZZY1 grains was higher than that in ZZY8 except for the heading stage, and the largest difference between varieties was observed at maturity (Figure 8b). Regarding starch transport rate, both varieties exhibited a trend of increasing first and then decreasing. The proportion of starch to nonstructural carbohydrates in ZZY1 was higher than that in ZZY8 from 10 DAH to maturity (Figure 8b).

**Figure 9:**
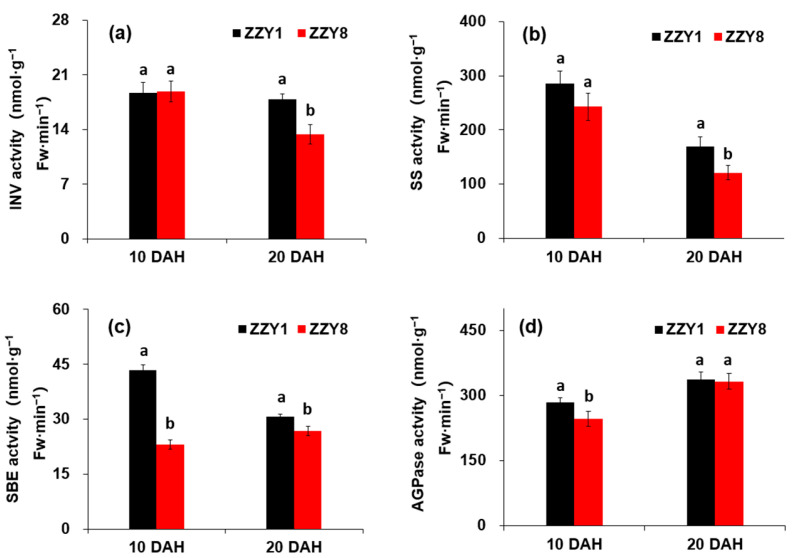
The activities of key enzymes related to grain-filling in grains of rice. (**a**) Invertase (INV) activity; (**b**) soluble sucrose synthase (SS); (**c**) starch branching enzyme (SBE) activity; (**d**) ADP-glucose pyrophosphorylase (AGPase) activity. DAH denotes days after heading; ZZY1 and ZZY8 indicate rice varieties, Zhongzhouyou1 and Zhongzhouyou8, respectively; error bars denote ± standard deviation (*n* = 3); different letters between varieties at the same determination time indicate significant differences at the 0.05 probability level.

The activities of enzymes, including invertase, SS, SBE, and AGPase, were determined at 10 DAH and 20 DAH (Figure 9). The grain acid invertase content of both varieties did not show a significant difference at 10 DAH, whereas it was significantly lower in ZZY8 than ZZY1 at 20 DAH, with a decrease of 25.0% (Figure 9a). Compared to 10 DAH, the SS activity in the grains of ZZY1 was significantly reduced, while it was slightly increased in ZZY8 at 20 DAH (Figure 9b). However, the SS activity of ZZY8 was significantly lower than that of ZZY1 at both determination dates. The starch branching enzyme activity of ZZY1 grains was significantly higher than ZZY8 at 10 DAH, while the difference between the two varieties was not significant at 20 DAH (Figure 9c). The AGPase activity in ZZY1 grains was higher than that of ZZY8 regardless of the determination date (Figure 9d). The difference between varieties was not significant at 10 DAH, while it was significantly higher in ZZY1 than in ZZY8 at 20 DAH.

### 2.7. Characteristics of Energy Metabolism

**Figure 10:**
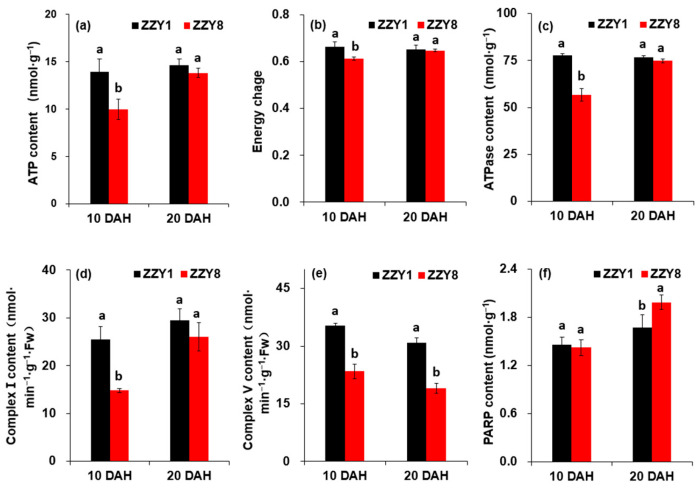
Energy allocation in grains of rice. (**a**) ATP content; (**b**) energy charge; (**c**) ATPase content; (**d**) Complex I content; (**e**) Complex II content; (**f**) PARP content. DAH denote days after heading; ZZY1 and ZZY8 indicate rice varieties, Zhongzhouyou1 and Zhongzhouyou8, respectively; error bars denote ± standard deviation (*n* = 3); different letters between varieties at the same determination time indicate significant differences at the 0.05 probability level.

The ATP, ATPase content, and energy charge values of ZZY1 grains were significantly higher than those of ZZY8 at 10 DAH, while the difference between the varieties was not significant at 20 DAH (Figure 10a–c). The activity of the respiratory electron transport chain complex I in ZZY1 was significantly higher than in ZZY8 at 10 DAH, whereas the difference was not significant at 20 DAH (Figure 10d). The activity of the respiratory electron transport chain complex V in ZZY1 was significantly higher than in ZZY8 at both 10 and 20 DAH (Figure 10e). The PARP content in ZZY8 was only significantly higher than in ZZY1 at 20 DAH (Figure 10f).

### 2.8. Transcriptome Analysis of Carbohydrate and Energy Metabolism

**Figure 11:**
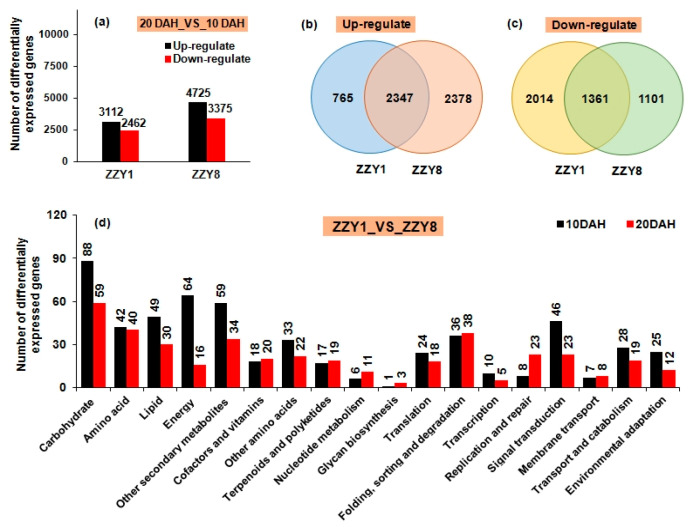
The transcriptome analysis of differentially expressed genes in grains of rice. (**a**) Number of differentially expressed genes at 20 days after heading (DAH) compared with 10 DAH in two varieties, with significantly different expression levels between different comparison groups; (**b**) the overlap of genes exhibiting significant upregulated expression at 20 DAH compared with 10 DAH; (**c**) the overlap of genes exhibiting significant downregulated expression at 20 DAH compared with 10 DAH; (**d**) Kyoto Encyclopedia of Genes and Genomes (KEGG) pathway annotation of differentially expressed genes in ZZY1 compared with ZZY8, respectively, at 10 DAH and 20 DAH.

Transcriptomic studies were used to investigate the role of energy in yield and quality formation. The number of differentially expressed genes at 20 DAH compared with 10 DAH in ZZY8 was significantly higher than that in ZZY1. The upregulated gene number was 4725 and the downregulated number was 3375 in the former, while the numbers were 3112 and 2462, respectively, in the latter (Figure 11a). This indicated that more intense metabolic changes occurred in the grains of ZZY8 at 20 DAH, whereas the changes in ZZY1 were relatively small. There were 2347 commonly upregulated genes shared by the two varieties (Figure 11b), and the functions of these genes mainly focused on stress response, abscisic acid response, dehydration response, and protein modification (Figure S1). There were 1361 commonly downregulated genes (Figure 11c), and they were mainly related to carbohydrate and energy metabolism, cellular function, and structure (Figure S2). This indicated that grain-filling at 20 DAH tended to slow down and inner metabolic function activities had reduced. Transcriptome analysis of both varieties at the same determination time indicated that the expression of differentially expressed genes was significantly higher at 10 DAH than at 20 DAH (Figure 11d). At 10 DAH, the greatest changes included carbohydrate metabolism, energy metabolism, and secondary metabolism, while the affected pathways were carbohydrate metabolism, amino acid metabolism, and secondary metabolism pathways at 20 DAH.

**Figure 12:**
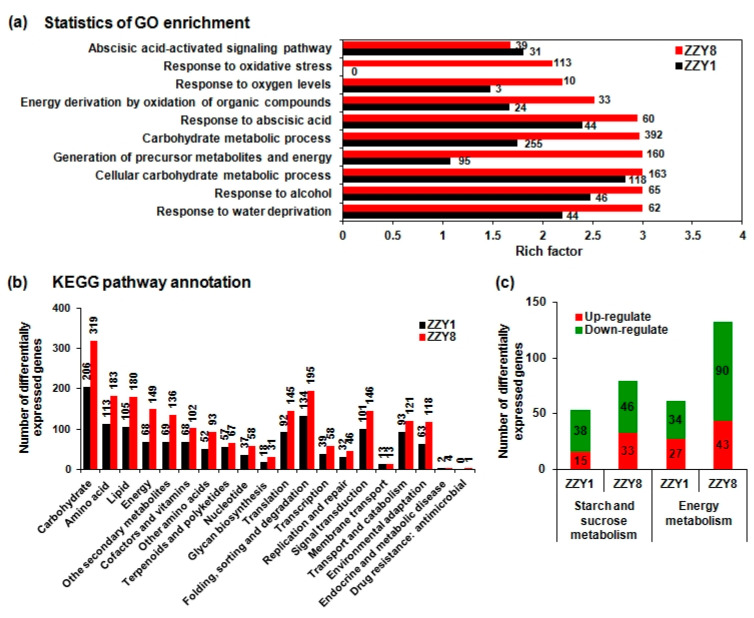
Distribution of genes with statistically changed expression levels in grains of two rice varieties. (**a**) Gene ontology (GO) enrichment analysis of the differentially expressed genes at 20 days after heading (DAH) compared with 10 DAH in two varieties; (**b**) Kyoto Encyclopedia of genes and genomes (KEGG) pathway annotation of the differentially expressed genes at 20 DAH compared with 10 DAH in two varieties; (**c**) differential expression gene analysis of starch and sucrose metabolism and energy metabolism pathways. ZZY1 and ZZY8 indicate rice varieties, Zhongzhouyou1 and Zhongzhouyou8, respectively.

Gene ontology enrichment analysis of differentially expressed genes shows that these genes were primarily related to abscisic acid signaling, dehydration response, carbohydrate metabolism, and energy metabolism (Figure 12a). The Kyoto Encyclopedia of Genes and Genomes pathway annotations for the differentially expressed genes on different days after flowering for both varieties show that these genes were mainly related to carbohydrate, amino acid, and energy metabolism pathways, as well as protein processing and environmental response genes (Figure 12b). Additionally, increased responses to oxygen content and ethanol suggested an enhancement of anaerobic respiration metabolism in the grains at 20 DAH. Analysis of differentially expressed genes involved in starch and sucrose metabolism, as well as energy metabolism pathways (glycolysis, TCA, oxidative phosphorylation), revealed that genes associated with starch metabolism and energy metabolism were significantly downregulated at 20 DAH, with the number of downregulated genes in ZZY8 being greater than in ZZY1, indicating intense inhibition of starch metabolism and energy metabolism in ZZY8 (Figure 12c).

## 3. Discussion

The results of this experiment indicate that both the actual and theoretical yield of ZZY1 under field conditions were higher than ZZY8, with increases of 17% and 7.84%, respectively (Figure 1). Although there have been many studies regarding the yield performance and growth characteristics in rice varieties of ZZY1 or ZZY8, there were few research results specialized in comparing the two varieties. According to the yield components, ZZY1 had higher effective panicle numbers, seed-setting rate, and grain weight when compared to ZZY8, in which the increment in seed-setting rate was 11.4% (Figure 1a,c,d). Therefore, the higher seed-setting rate was considered the main reason for the significantly higher grain yield of ZZY1 than ZZY8.

It is well known that the yield formation of rice is closely associated with the production, accumulation, and translocation of assimilates [1,25]. However, the higher yield of ZZY1 does not seem to be highly related to its photoassimilates accumulation. There were no significant differences in leaf chlorophyll content and net photosynthetic rate between the two varieties (Figure 4), and the total dry matter of ZZY1 was lower than ZZY8, especially during maturity (Figure 5a). In this experiment, the difference in spikelet numbers per panicle between the two varieties was small, but ZZY1 had a higher seed-setting rate and grain weight than ZZY8, indicating that the lower yield of ZZY8 was mainly attributable to inefficient assimilate transport. Similar research has been reported by Zhang et al. [26], in which it was indicated that the decrease in grain weight caused by high temperature during flowering was mainly due to inefficient transport or assimilate translocation rather than source and sink limitations. Furthermore, this was confirmed by the significant differences in the proportion of dry matter of the panicle to that of the total between the two varieties (Figure 5b). Additionally, the proportion of nonstructural carbohydrates in the panicles to the total in ZZY1 was higher than ZZY8 (Figure 6d). Therefore, higher assimilate transport and distribution are the main reasons for the higher yield of ZZY1.

With the improvement of living standards in recent years, high-yield and high-quality have become the primary goals of rice breeding. To improve rice quality while ensuring a certain grain yield has become a consensus in modern rice breeding [13,14,15]. Based on the analysis above, ZZY1 has a higher grain yield than ZZY8, while the latter has better rice quality (Figure 2). There have been many reports on the trade-off between grain yield and rice quality, but the underlying mechanisms have not been elucidated. It is believed that this may be related to energy production and utilization. Studies have shown that the formation of grain yield and rice quality is an energy-consuming process [21,22,23]. Insufficient energy or low utilization efficiency makes it difficult to meet the demands for both yield and quality. It was observed that the ATP content, ATPase activity, and energy charge of the grains of ZZY1 were higher than ZZY8 at 10 days after heading (DAH), while there was almost no difference between the two varieties at 20 DAH (Figure 10). This might be one of the main causes of the lower quality in ZZY1 compared to ZZY8.

Numerous studies have shown that the improper dynamics of grain filling and changes in the physiological activity of related key enzymes have a significant impact on chalkiness formation [27,28]. Enzyme activities in regions prone to form chalkiness are relatively low, resulting in an imbalance in starch synthesis and accumulation [29,30,31]. Varieties with a smooth grain-filling dynamic and a relatively slow grain-filling rate often have less chalkiness, while those with a fast-filling rate or large fluctuations in grain-filling dynamics tend to have more chalkiness [32]. In this experiment, ZZY1 had a significantly higher grain-filling rate than ZZY8, while it exhibited large fluctuations in grain-filling rate. The grain-filling rate of ZZY1 showed a rapid rise and then a sharp fall, and this might be the main reason for its significantly higher chalkiness compared to ZZY8 (Figure 7b). Starch synthesis in grains mainly depends on a series of enzymes such as AGPase, granule-bound starch synthase (GBSS), SS, SBE, and starch debranching enzyme (DBE), and changes in starch metabolism enzyme activity are closely related to chalkiness formation [33,34,35]. AGPase catalyzes the initiation of starch synthesis, in which ATP and 1-phosphate glucose are converted into ADP-glucose, transported to starch granules by the BT1 transporter protein on the starch body, and then synthesized into starch by GBSS, SS, and other enzymes [36]. It is the rate-limiting enzyme in starch synthesis, and the activity of AGPase is significantly correlated with grain-filling rate [25]. It was observed that the key enzyme activity of grain-filling in ZZY1 was higher than ZZY8 at 10 or 20 days after heading (DAH), which may be an important reason for the higher grain-filling rate of ZZY1 than ZZY8 (Figure 9). However, at the same time, the increase in grain-filling enzyme activity consumed a lot of energy, resulting in insufficient energy required for other indices of rice quality, which ultimately affected the formation of rice quality. This may also be an important reason for the increase in grain weight but the deterioration of the quality of ZZY1. Both the carbohydrate and energy metabolism differences between varieties were inferred at the gene expression level (Figure 11 and Figure 12). The results of the current study give us the inspiration that cultivation measures such as nitrogen management or exogenous chemical spraying should be used by regulating energy metabolism [24,37]. Furthermore, the indices related to energy distribution and utilization were not determined in this experiment. Thus, the underlying mechanism of energy metabolism involved in grain yield and rice quality formation remains to be clarified. Research concerning breeding and cultivation methods to simultaneously improve grain yield and rice quality by enhancing energy supply is needed in future studies.

## 4. Materials and Methods

### 4.1. Materials and Experimental Design

The experiment was conducted in 2020 and repeated in 2021 at the Fuyang base of the China National Rice Research Institute (30.30′ N, 120.2′ E, 11 m above sea level). Two three-line *indica* hybrid rice varieties, ZZY1 (Zhongzhe A/Hanghui 570), and ZZY8 (Zhongzhe A/T-8), that are suitable for planting in the middle and lower reaches of the Yangtze River, were used as the study materials in the field experiments. The whole growth period of ZZY1 is approximately 137 days, while that of ZZY8 is slightly longer, approximately 159 days. Both of them were widely used in local production because of their high yield performances, good rice quality characteristics, and high stress resistances than other varieties. The full heading date of ZZY1 and ZZY8 was approximately 15–18 August and 4–6 September across two experimental years. After soaking the rice seeds for 48 h, they were germinated in a constant temperature box at 37 °C for 24 h, and then sown on the seedbed on 21 May. Transplanting was carried out after 25 days, on 15 June.

**Figure 13:**
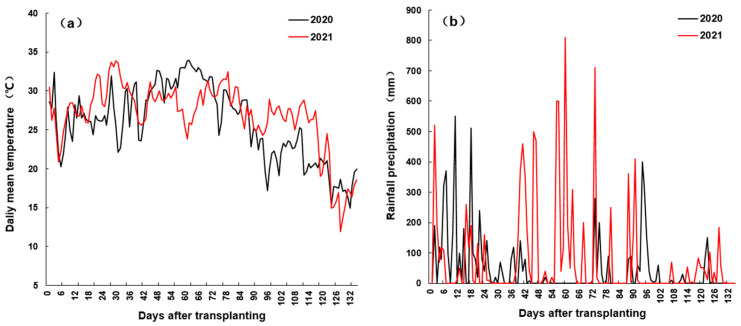
The daily mean air temperatures (**a**) and rainfall precipitation (**b**) during the rice growing season from June to October of 2020 and 2021 at the experimental site of Fuyang, Hangzhou.

The test field was a loamy clay soil that contained 37.2 g kg^−1^ organic matter, 305.8 mg kg^−1^ alkali-hydrolyzable nitrogen, 20.6 mg kg^−1^ available phosphorus, and 68.9 mg k^−1^ available potassium, with pH 5.79. The area of each experimental plot was 25 m^2^, with a transplanting row spacing of 20 cm × 25 cm, and each variety was replicated in three plots with a complete randomized block design. Field management followed the conventional high-yield cultivation practices for rice production. Nitrogen was applied with a total amount of 195 kg ha^−1^ in the form of urea, respectively, before transplant, at midtillering, and panicle initiation as topdressing with a proportion of 5:2:3. Phosphorus was applied in the amount of 50 kg ha^−1^ before transplanting in the form of calcium magnesium phosphate composite fertilizer. Potassium was incorporated into the experimental plots at the rate of 40 kg ha^−1^ both before transplanting and at midtillering. The paddy field was kept with a shallow water layer of approximately 2 cm until one week before harvest, except for drainage at the end of tillering. Periodic manual weeding and spraying to control pests and disease were carried to guarantee healthy growth of rice plants. The average daily air temperatures and rainfall precipitation during the rice growing season from June to October of the two study years obtained from the weather station near the experimental site are shown in Figure 13.

### 4.2. Grain Yield, Its Components, and Rice Quality Determination

The rice plants were harvested after maturity. Twelve hills were sampled from each plot, and the yield was calculated by investigating the number of effective panicles per plant, the number of grains per panicle, seed-setting rate, and 1000-grain weight. The actual harvest from 10 m^2^ area of the field trial plots was also recorded and converted into a moisture content of 13.5%. Quality testing was conducted after drying, with samples sent to the Rice Quality Testing Center of the China National Rice Research Institute, Chinese Academy of Agricultural Sciences.

### 4.3. Plant Height and Tiller Dynamics

Plant height and tiller numbers were surveyed every week from 7 to 63 days after transplanting. Observation points were selected in each replicate plot, and ten hills were determined at each point.

### 4.4. Dry Matter Production and Accumulation

Six hills of rice plants were randomly sampled from each plot at the tillering, panicle differentiation, heading, 10 days after heading (DAH), 20 DAH, and maturity stages. The plants were then divided into stem sheath, leaves, and panicles, and dried at 105 °C for 60 min and then at 80 °C for 48 h until a constant weight was reached in an oven.

### 4.5. Carbohydrate Content Determination

The contents of soluble sugars and starch were determined using the anthrone colorimetric method [38]. A dried sample of 0.2 g was taken, added to 10 mL of deionized water, and boiled for 30 min in a constant temperature water bath. After cooling and centrifugation, the supernatant was collected. This extraction process was repeated three times, and the extracts were combined and quantified to a certain volume for the determination of soluble sugar content. The remaining residue was dried in an oven, added to 2 mL of deionized water, boiled in a water bath for 20 min, then 2 mL of 9.2 mol L^−1^ HClO_4_ was added. After cooling, 6 mL of deionized water was added, and the mixture was centrifuged at 2000 rpm for 20 min to collect the supernatant. The remaining residue was again treated with 2 mL of 4.6 mol L^−1^ HClO_4_, and the process was repeated. Both extracts were then combined for starch content determination. The nonstructural carbohydrates (NSC) are the total of soluble sugar and starch contents.

### 4.6. Grain-Filling Dynamics

Two hundred rice panicles that flowered on the same day and grew evenly were selected and tagged to mark the flowering date. From flowering to maturity, the marked panicles were sampled every 3 days in the early grain-filling stage and every 6 days in the later grain-filling stage to investigate the weight of superior and inferior grains. The grain-filling process was fitted using the Richards’ growth equation W = A/(1 + Be^−kt^)^1/N^ to calculate the corresponding filling rates [39,40]. In this equation, A indicates the final growth weight (mg) of a grain calculated by the growth equation, while B, k, and N are coefficients determined by regression analysis. R^2^ is the coefficient of determination.

### 4.7. Key Enzyme Activities During Grain-Filling

Rice grains were sampled 10 and 20 days after heading (DAH) and stored at −80 °C for the determination of various starch synthesis-related enzyme activities. Approximately thirty grains were removed from the shells, and 5 mL of 100 mmol L^−1^ Tricine-NaOH extraction solution [pH 8.0, containing 10 mmol L^−1^ MgCl_2_, 2 mmol L^−1^ EDTA, 50 mmol L^−1^ β-mercaptoethanol, 12% (*v*/*v*) glycerol, 5% (*w*/*v*) PVP 40] was added. The mixture was ground on ice, centrifuged at 15,000× *g* for 5 min (4 °C), and the supernatant (crude enzyme solution) was collected following the method by Li et al. [41] with slight modifications. The activities of ADP-glucose pyrophosphorylase (AGPase), sucrose synthase (SS), and starch branching enzyme (SBE) are expressed in nmol g^−1^ Fw min^−1^.

Another set of grains were finely powdered in liquid nitrogen, followed by homogenization in the 20% extraction buffer comprising 50 mmol L^−1^ HEPES (pH 7.5), 5 mmol L^−1^ MgCl_2_, 1 mmol L^−1^ EDTA-Na_2_, 0.5 mmol L^−1^ dithiothreitol, 1% Triton X-100, 2% polyvinyl pyrrolidone, and 10% glycerol. The resulting supernatant was promptly desalted using a Sephadex G-25 medium, pre-equilibrated with the extraction buffer at 4 °C. The filtered solution was promptly utilized to assess the activity of soluble acid invertase by using the test kit provided by Comin Biotechnology Co., Ltd. (Suzhou, China).

### 4.8. Determination of Energy Metabolism Related Indicators

ATP, ATPase, ADP, AMP, and poly-ADP-ribose polymerase (PARP) were all determined using a double-antibody sandwich ELISA method. The coated microplates with plant ATP, ATPase, ADP, AMP, and PARP capture antibodies were sequentially added with specimens, standards, and HRP-labeled detection antibodies, followed by incubation and thorough washing. Substrate TMB turned blue under the catalysis of peroxidase and finally turned yellow under acidic conditions. The intensity of the color is directly proportional to the amounts of ATP, ATPase, ADP, AMP, and PARP in the samples. Absorbance was measured at 450 nm wavelength using a Multiskan FC ELISA reader (Thermo Fisher Scientific (Shanghai) Instrument Co., Ltd., Shanghai, China). The specific operations were completed following the related reagent kit manual (Shanghai Enzyme-linked Biotechnology Co., Ltd., Shanghai, China).

Mitochondrial complex I, complex IV, and complex V were determined as referring to the instructions of the reagent kits (Suzhou Comin Biotechnology Co., Ltd., Suzhou, China).

### 4.9. RNA-Seq and Bioinformatics Analysis

The Plant RNA Purification Reagent was used to extract total RNA from the grains, and genomic DNA was further removed by DNase I (TaKaRa Biotechnology, Dalian, China). The TruSeq^TM^ RNA sample preparation kit (Illumina, San Diego, CA, USA) was used to construct the library. After quantification using TBS-380 (Beijing Yuanpinghao Biotechnology Co., Ltd., Beijing, China), the library was sequenced on the Illumina/NovaSeq 6000 sequencing platform with a reading length of PE150. Transcriptome sequencing and analysis services were provided by Shanghai Majorbio Bio-Pharm Technology Co., Ltd., Shanghai, China.

### 4.10. Statistical Analysis

Data from the experiment were statistically analyzed using Excel and SPSS software version 11.5 (IBM Corp., Armonk, NY, USA).

## 5. Conclusions

The grain yield of ZZY1 was higher, while the rice quality was poorer than that of ZZY8. The high yield of ZZY1 was mainly benefited from its higher seed-setting rate and grain weight. Correspondingly, the proportion of panicle dry matter weight or nonstructural carbohydrate to the total of ZZY1 was higher than that of ZZY8. The maximum grain-filling rate, average grain-filling rate, starch contents, and key enzyme activities involved in starch synthesis in the former were significantly higher than those of the latter. Furthermore, the ATP/ATPase content, ATP metabolism-related enzyme activity, and energy charge values in the grains of ZZY1 were higher than those of ZZY8 at the early grain-filling stage. The above results showed that carbohydrate and energy metabolism were the main ways affecting the yield and quality of the two varieties. It was verified by the transcriptome analysis, demonstrating that the energy production of ZZY1 was insufficient to simultaneously supply the needs thus leading to the discordant formation in its grain yield and rice quality formation.

## Figures and Tables

**Table 1:** The average grain-filling rate, the maximum grain-filling rate, and the active grain-filling period.

Variety/Grain Type	Mean Grain-Filling Rate (g·grain ^−1^·d ^−1^)	Active Grain-Filling Period (d)	Period to Reach Maximum Grain-Filling Rate (d)	Maximum Grain-Filling Rate (g·grain ^−1^·d ^−1^)
ZZY1-Superiors	2.33 a	10.6 d	3.9 c	2.54 a
ZZY1-Inferiors	1.04 c	23.7 b	15.9 a	1.14 b
ZZY8-Superiors	1.63 b	14.5 c	3.7 c	2.01 a
ZZY8-Inferiors	0.79 d	30.8 a	14.3 b	1.11 b

ZZY1 and ZZY8 indicate the rice varieties Zhongzheyou1 and Zhongzheyou8, respectively. Error bars denote ± standard deviation (*n* = 3). Different letters in the same column indicate significant differences at the 0.05 probability level.

## Data Availability

The data that support the results of this study are available from the corresponding author, upon reasonable request.

## Supplementary Materials

The supporting information can be downloaded at: https://www.mdpi.com/article/10.3390/ijms252312751/s1.

## Abbreviations

AGPase: ADP-glucose pyrophosphorylase; DAH, days after heading; DBE, starch debranching enzyme; GBSS, granule-bound starch synthase; NSC, non-structural carbohydrates; SBE, starch branching enzyme; SS, sucrose synthase; ZZY1, Zhongzheyou1; ZZY8, Zhongzheyou8.
