# Supplementary material for: Energy Deficiency and Misdistribution Leads to Disrupted Formation in Grain Yield and Rice Quality
Source: Int J Mol Sci. 2024 Nov 27;25(23):12751. doi: 10.3390/ijms252312751 (PMC11640994; doi:10.3390/ijms252312751)
Supplement: Supplementary file 1 [file ijms-25-12751-s001.zip › ijms-3316460-supplementary.pdf]

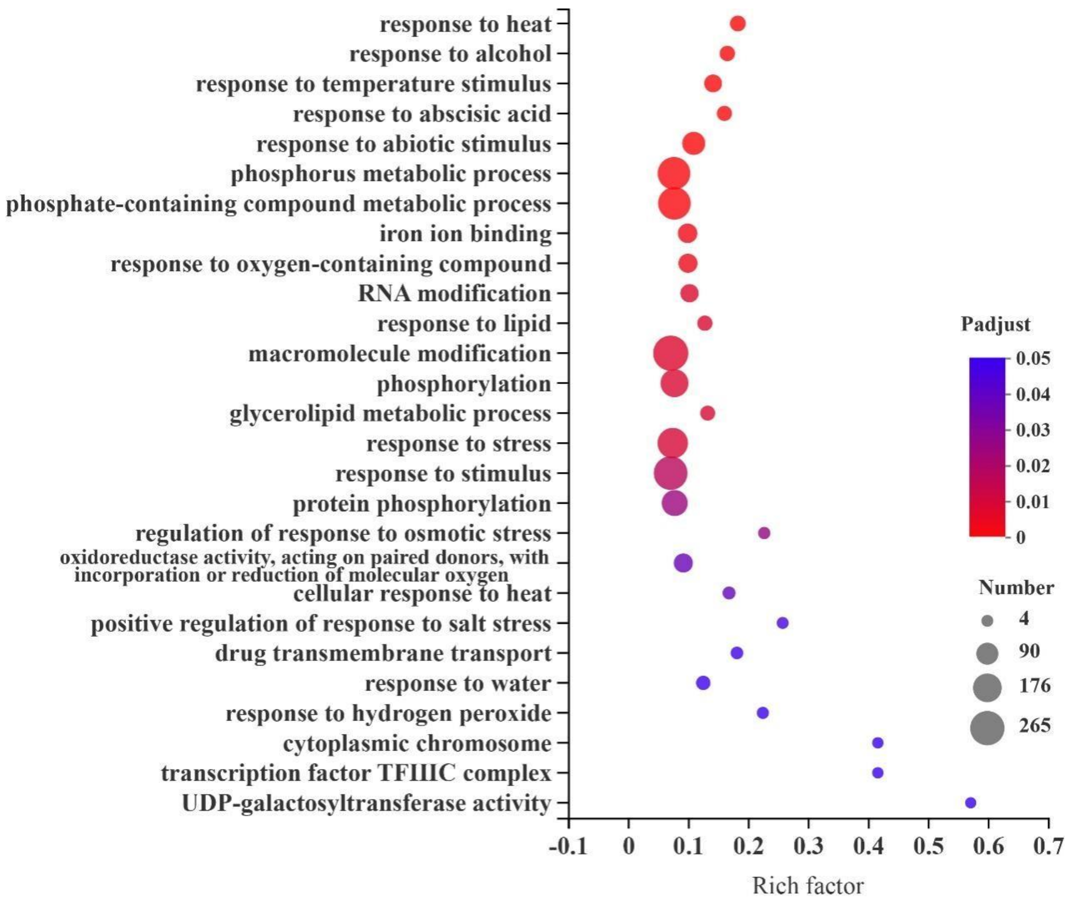

Figure S1. Gene ontology (GO) enrichment analysis of the genes jointly up-regulated in Zhongzhouyou8 and Zhongzhouyou1 at 10 days after heading compared with 20 days after heading.

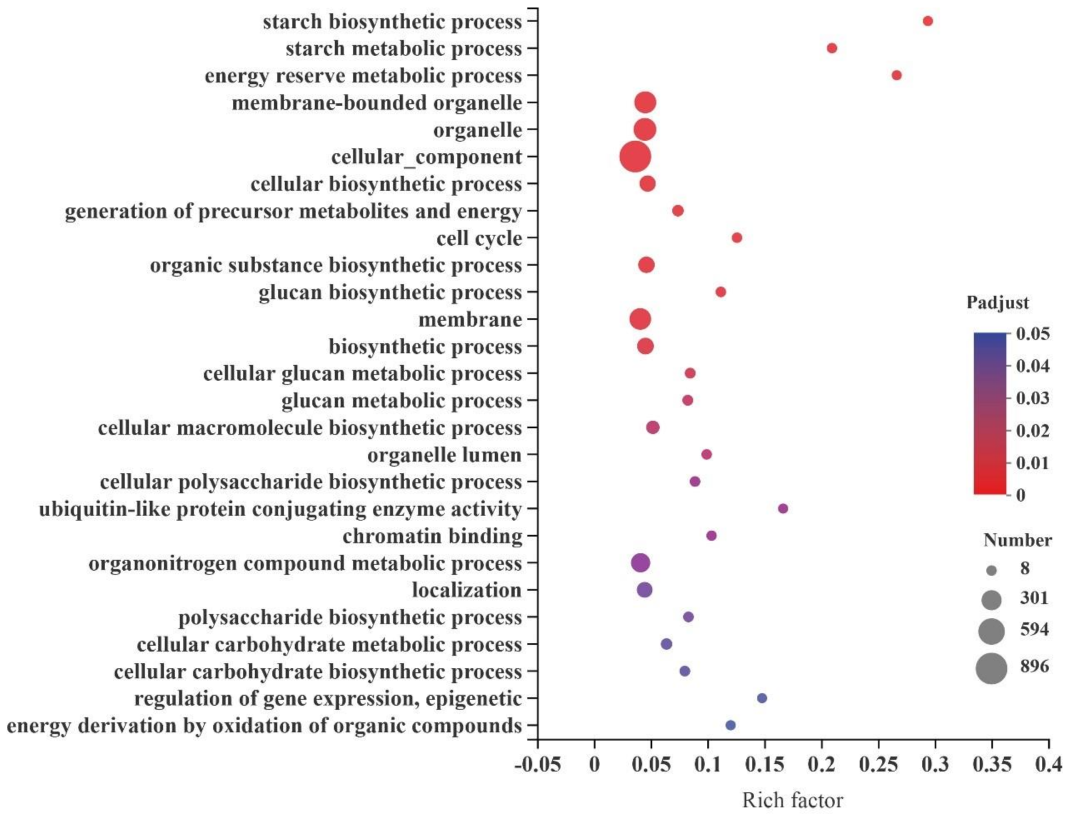

Figure S2. Gene ontology (GO) enrichment analysis of the genes jointly down-regulated in Zhongzhouyou8 and Zhongzhouyou1 at 10 days after heading compared with 20 days after heading.
